# Metabolites of *Xenorhabdus* bacteria are potent candidates for mitigating amphibian chytridiomycosis

**DOI:** 10.1186/s13568-023-01585-0

**Published:** 2023-08-24

**Authors:** János Ujszegi, Zsófia Boros, András Fodor, Balázs Vajna, Attila Hettyey

**Affiliations:** 1 Department of Evolutionary Ecology, Plant Protection Institute, Centre for Agricultural Research, Eötvös Loránd Research Network, Budapest, Hungary; 2 Department of Systematic Zoology and Ecology, Eötvös Loránd University, Budapest, Hungary; 3 Department of Genetics, Eötvös Loránd University, Budapest, Hungary; 4 Department of Microbiology, Eötvös Loránd University, Budapest, Hungary

**Keywords:** Amphibian pathogen, Antifungal treatment, Antimicrobial, Bioaugmentation, Chemical-free disinfection, Skin microbiota

## Abstract

Chytridiomycosis, caused by the chytrid fungus *Batrachochytrium dendrobatidis* (Bd), has caused extreme losses in amphibian biodiversity. Finding bacteria that produce metabolites with antifungal properties may turn out to be invaluable in the fight against this devastating disease. The entomopathogenic bacteria, *Xenorhabdus szentirmaii* and *X. budapestensis* produce secondary metabolites that are effective against a wide range of fungal plant pathogens. To assess whether they may also be effective against Bd, we extracted cell-free culture media (CFCM) from liquid cultures of *X. szentirmaii* and *X. budapestensis* and tested their ability to inhibit Bd growth in vitro. As a second step, using juvenile common toads (*Bufo bufo*) experimentally infected with Bd we also tested the in vivo antifungal efficacy of *X. szentirmaii* CFCM diluted to 2 and 10% (v/v), while also assessing possible malign side effects on amphibians. Results of the in vitro experiment documented highly effective growth inhibition by CFCMs of both *Xenorhabdus* species. The in vivo experiment showed that treatment with CFCM of *X. szentirmaii* applied at a dilution of 10% resulted in infection intensities reduced by ca. 73% compared to controls and to juvenile toads treated with CFCM applied at a dilution of 2%. At the same time, we detected no negative side effects of treatment with CFCM on toad survival and development. Our results clearly support the idea that metabolites of *X. szentirmaii*, and perhaps of several other *Xenorhabdus* species as well, may prove highly useful for the treatment of Bd infected amphibians.

**Supplementary Information:**

The online version contains supplementary material available at 10.1186/s13568-023-01585-0.

## Key points

1. First report of high anti-Bd efficacy of *Xenorhabdus* metabolites in vitro.
2. Metabolites of *X. szentirmaii* can be applied effectively on live toads.
3. Application of *X. szentirmaii* metabolites on live toads was safe.

**Supplementary Information:**

The online version contains supplementary material available at 10.1186/s13568-023-01585-0.

## Introduction

Amphibians have suffered rapid biodiversity loss over the last decades and became one of the most threatened vertebrate groups (Monastersky 2014). The main causes of this decline are climate change, pollution, habitat loss, and emerging infectious diseases (Wake and Vredenburg 2008; Hof et al. 2011; Campbell Grant et al. 2016). Chytridiomycosis is the most serious emerging disease affecting amphibians caused by the chytrid fungi *Batrachochytrium dendrobatidis* (Bd) and *Batrachochytrium salamandrivorans* (Bsal). This disease has already led to the decline or extinction of several hundred species and continues to cause mass mortality events on five continents (Scheele et al. 2019). Because Bsal has only been discovered recently (Martel et al. 2013) and its distribution range is confined to date to the northwest of continental Europe (Spitzen-van der Sluijs et al. 2016; González et al. 2019), here we concentrate on the better known and globally distributed Bd. The fungus infects keratinous epidermal layers of the skin (Berger et al. 1998). The symptoms of the disease are therefore the intensive sloughing or skin shedding, reddening on legs and ventral surfaces even ulcerations or skin lesions. The structural damage of the skin can impair skin-breathing and osmoregulation, provoking shifts in electrolyte balance and finally leading to cardiac asystolic death in metamorphosed amphibians (Voyles et al. 2009). Since its global appearance, several countermeasures against chytridiomycosis have been proposed (Johnson et al. 2003; Woodhams et al. 2003; Harris et al. 2006; Woodward et al. 2014; Hettyey et al. 2019) but finding a widely applicable mitigation method has remained one of the most challenging goals of animal conservation (Garner et al. 2016; Scheele et al. 2019).

Amphibians have a broad repertoire for defence against pathogens via thermoregulatory behaviour (behavioural fever; Sherman et al. 1998; Richards-Zawacki 2010; Murphy et al. 2011) and their highly developed adaptive and innate immune systems (Carey et al. 1999; Grogan et al. 2018). Intrinsic factors of the adaptive immune system, such as the major histocompatibility complex class II (MHCII) can play important roles in determining the susceptibility of the host to pathogens (Barribeau et al. 2008; Bataille et al. 2015; Savage and Zamudio 2016). The innate immune system consists of cells with the function of absorbing and presenting antigens to the adaptive immune system. The complement system (humoral part of the innate immune system) leads the chemotaxis of phagocytes and aids the penetration of prokaryote and fungal cell membranes (Carey et al. 1999; Speth et al. 2008). However, the first line of defence against invading pathogens are the skin-secreted defensive chemicals such as antimicrobial peptides, steroids, alkaloids, and biogenic amines (Daly 1995; Macfoy et al. 2005; Gomes et al. 2007; Tempone et al. 2007; König et al. 2015), and mutualistic skin bacteria, that can prevent infections or disease propagation (Belden and Harris 2007; Krynak et al. 2016). Nonetheless, while their natural defence mechanisms are normally effective against pathogens and parasites, emerging infectious diseases caused by introduced pathogens can have devastating effects on amphibian populations, especially when they act in concert with other stress factors (Koprivnikar 2010; Campbell Grant et al. 2016).

Bioaugmentation, which is the restoration or enrichment of the microbiota to provide additional defences against pathogens, has been found to be useful in agriculture (Patterson and Burkholder 2003; Gentry et al. 2004), aquaculture (Olsson et al. 1992), and in the conservation of corals (Teplitski and Ritchie 2009). Addition or supplementation of mutualistic skin bacteria could be a promising method to mitigate the impact of chytridiomycosis as well (Harris et al. 2009; Bletz et al. 2013; Rebollar et al. 2020). As described in natural habitats, the presence of certain microbial taxa (e.g., species of *Janthinobacterium*, *Lysobacter*, *Pseudomonas* genera) may enhance population resistance to chytridiomycosis in some amphibian species (Woodhams et al. 2007; Lam et al. 2010; Walke et al. 2011; Rebollar et al. 2016a). Bacterial secondary metabolites produced by these microbes associated with the amphibian skin inhibited Bd growth effectively in vitro (Woodhams et al. 2007; Brucker et al. 2008a, b; Myers et al. 2012). Furthermore, the presence of some of the above mentioned bacterial taxa also reduced infection intensities and enhanced the survival of animals in vivo (Becker et al. 2009; Muletz et al. 2012). However, other studies delivered mixed results reporting moderate, or no effect of bioaugmentation (Woodhams et al. 2012; Küng et al. 2014; Rebollar et al. 2016b). Research on bioaugmentation against chytridiomycosis mainly focused on the establishment of certain bacteria producing antifungal compounds or entire bacterial communities on the amphibian skin (Rebollar et al. 2020). However, this approach also has several limitations. The introduction of new bacteria can induce disadvantageous changes in the hosts’ microbiome, they can trigger immune responses in hosts, and environmental conditions varying across time and space can differentially affect microbial community structure and function, and the effectiveness of defence against pathogens (Daskin et al. 2014; Robak and Richards-Zawacki 2018; Woodhams et al. 2018; Rebollar et al. 2020). Additionally, the presence of Bd can also change the structure of the skin microbial community, possibly by suppressing the growth of the beneficial bacteria (Jani and Briggs 2014; Woodhams et al. 2018). Furthermore, the introduction of bacteria to ecosystems where they have not been present before can be hazardous (Simberloff et al. 2013).

Utilizing bacterial metabolites directly against Bd instead of trying to establish live cultures on amphibian hosts has also been tested (Bell et al. 2013; Madison et al. 2017). Without the need for the establishment of live cultures on amphibian skin, this approach may be applied more easily and safely than bioaugmentation. Treatment can be safely controlled and better standardized because the amount of antifungal metabolites can be adjusted so as not to be harmful to amphibians. Finally, the scope of the search for antifungal metabolites with broad-spectrum inhibition capabilities can be widened to cover novel microbial sources, even of non-amphibian origin.

The idea of using wide-spectrum antimicrobials produced by the entomopathogenic nematode-bacterium (EPN-EPB) symbiotic complexes (Akhurst 1982) was first suggested by Bode (2009). Entomopathogenic nematode species belonging to the *Steinernema* and *Heterorhabditis* genera are parasites of soil-dwelling insects. Their infective dauer juveniles (IJ, Kaya 1978), carry cells of species-specific obligate entomopathogenic bacterial (EPB) symbionts. Right after the IJ enters the insect body cavity, it releases the EPBs into the hemocoel, where the bacteria start to propagate and synthesize efficient insecticide toxins and various secondary metabolites which suppress the host’s immune response, and accelerate its death. The symbiotic bacteria also produce antimicrobial metabolites which protect the cadaver against microbial food competitors (Forst et al. 1997) keeping pathobiome conditions (Ogier et al. 2020) favourable for the EPN-EPB symbiotic complex in the cadaver and ambient soil. *Xenorhabdus szentirmaii* and *X. budapestensis* (Lengyel et al. 2005), the natural symbionts of the EPN species *Steinernema rarum* (de Doucet 1986) and *S. bicornutum* (Tallósi et al. 1995) respectively, seem to be the two most potent EPB strains against bacterial, fungal, and protozoan pathogens of plants, livestock, and even humans (Furgani et al. 2008; Böszörményi et al. 2009; Vozik et al. 2015; Wenski et al. 2020; Fodor et al. 2022). Of the antimicrobial peptides (AMPs) produced by these *Xenorhabdus* species, fabclavine (Fuchs et al. 2012, 2014) and its metabolic derivatives (Wenski et al. 2019, 2020; Watzel et al. 2021) exhibit exceptional antimicrobial potential against different targets (Cimen et al. 2021). This molecular arsenal in combination with the cheap and easy-to-handle cultivation under laboratory conditions, as well as the accessibility for genetic manipulations make *Xenorhabdus* species and their metabolites great candidates for the fight against diseases of amphibians, including chytridiomycosis.

In this study, we aimed to assess experimentally whether *Xenorhabdus szentirmaii* and *X. budapestensis* metabolites may be effective against Bd. We extracted cell-free culture media (CFCM) from their liquid cultures and assessed Bd growth inhibition capabilities in vitro as the practical first step towards finding the range of quantity required for the suppression of Bd. We also tested for possible toxic effects and treatment efficacy of *X. szentirmaii* CFCM in vivo on juvenile common toads (*Bufo bufo*) experimentally infected with Bd. Since the integrity of the skin microbiome is crucial for amphibian health and mitigation methods should not disrupt the microbial community present on the amphibian skin (Rebollar et al. 2020), we also observed possible changes in skin microbial community structure caused by treatments. Treatment with CFCM instead of inoculation with living bacterial cells allows to avoid the abovementioned problems arising from interactions with the host’s immune system and from the environment-dependence of the establishment and metabolite production of probiotic bacteria (Rebollar et al. 2020). Thus, the application of antifungal metabolites may minimise undesirable side-effects on the targeted as well as on non-target species and on ecosystem processes (Bletz et al. 2013).

## Methods

### Culturing of Xenorhabdus

We tested two previously described antimicrobial peptide (AMP)-producing EPB strains: *Xenorhabdus budapestensis* nov. DSM-16342T from Central Europe, and *X. szentirmaii* nov. DSM-16338T of South American origin, described in the Department of Genetics of Eötvös University Hungary and deposited in DSMZ, Braunschweig, Germany by Katalin Lengyel and her colleagues (Lengyel et al. 2005).

For culturing *Xenorhabdus*, we prepared Mueller–Hinton liquid medium (Mueller and Hinton 1941) by dissolving 21 g powder (obtained from Sigma-Aldrich, St. Louis, USA) in 1000 ml of distilled water and sterilized it by autoclaving at 121 °C for 15 min before use. We alternatively cultured *Xenorhabdus* species on Luria broth agar (LBA) plates flooded with Luria broth (LB) (10 g casein peptone, 5 g yeast extract, 10 g sodium chloride, and 17 g agar [LB and LBA, respectively] dissolved in 1000 ml distilled water) as described by Ausubel and colleagues (1999). Indicator plates (LBTA) were supplemented with bromothymol blue and 2,3,5-Triphenyltetrazolium chloride, and were used to distinguish AMP producing (phase I) and non-producing (phase II) variants (Leclerc and Boemare 1991). Fresh single phase I colonies derived from frozen bacterial stocks were used for each experiment as previously described (Furgani et al. 2008; Böszörményi et al. 2009; Vozik et al. 2015). Microbiological media were obtained from Biolab Zrt. (Budapest, Hungary).

We cultured EPBs in liquid TGhLY medium (mTGhLY; 8 g tryptone, 2 g gelatine-hydrolysate, 4 g lactose, and 5 g yeast extract in 1000 ml distilled water) with a 7-day old single colony grown on LBA. With the addition of yeast extract, we established this method to provide optimal growth conditions in the same media for both the EPB strains and Bd. In all other respects, this media is equivalent to the TGhL media used for the culturing of Bd (see below). Each *Xenorhabdus* culture in this study started with 5–10 ml of (either LB or Mueller-Hinton) liquid medium inoculated with a single colony of the respective bacterium picked from an LBTA indicator plate and incubated overnight at 28 °C in a water bath shaker (Lab-Line Orbital Shaker Water Bath, Marshall Scientific, USA). Each late-log phase inoculum was then added to 200 ml mTGhLY into 400 ml tissue culture flasks to create scale-up cultures.

### Preparation of cell-free culture media (CFCMs)

We incubated scale-up cultures of both *Xenorhabdus* species for 7 days at 25 °C on an orbital shaker platform (Gallencamp, UK) and centrifuged cultures at 6000 rpm for 20 min at 4 °C in 400 ml tubes using a JLA-10.500 type rotor (Avanti centrifuge J-26 XPI, Beckman Coulter, Indianapolis, USA). With these preparation conditions, production of antibiotic metabolites in *Xenorhabdus* cultures reaches a stationary phase in 5–6 days, containing the same amount of metabolites (Furgani et al. 2008; Böszörményi et al. 2009; Vozik et al. 2015). The supernatant was filtered through a sterile 0.22 μm nylon filter and centrifuged again at the same speed. We considered the resulting supernatant to be a cell-free culture medium (CFCM) of the antibiotic-producing *Xenorhabdus* strains and used it for bioassays. To confirm that CFCM-s were indeed cell-free, we diluted at least two replicates of each with sterile 2× LB, incubated them along with the experimental samples, and checked for bacterial growth on LBA plates. We stored CFCMs at 4 °C in glass bottles until further use.

### Maintaining and culturing of Bd

For all experiments, we used the global pandemic lineage (GPL) of Bd. The isolate (Hung_2014) originated from a *Bombina variegata* collected in 2014 by J. Vörös (Natural History Museum, Budapest, Hungary) in the Bakony Mountains, Hungary, and isolated by M.C. Fisher and colleagues (Imperial College London, London, UK). We maintained cultures in TGhL medium (mTGhL; 8 g tryptone, 2 g gelatine-hydrolysate, and 4 g lactose in 1000 ml distilled water) in 25 cm^2^ cell culture flasks at 4 °C and passaged them every three months into sterile mTGhL.

One week before the start of in vitro tests, we placed 2–2 ml of Bd stock cultures onto mTGhL agar plates (containing 1% agar w/v) in sterile plastic Petri dishes (90 mm diameter; Biolab Zrt) and incubated them at 20 °C for 7 days. Then we flooded the plates with 2 ml 1% tryptone medium (10 g tryptone in 1000 ml distilled water). After 30 min we collected the liquid media containing the zoospores and rinsed the plates with another 500 µl of 1% tryptone medium which we added to the previously obtained media. We estimated zoospore concentrations in the harvested media (also containing some zoosporangia) using a Bürker chamber at ×400 magnification and adjusted to 10^7^ zoospores (zsp)/ml in 1% tryptone medium.

One week before performing experimental infections in the in vivo experiment, we inoculated 100 ml mTGhLY with 2 ml of Bd stock culture in a 175 cm^2^ cell culture flask and incubated it for seven days at 21 °C. We assessed the concentration of intact zoospores using a Bürker chamber at ×400 magnification and diluted the zoospore suspension with mTGhLY to a final concentration of 10^6^ zsp/ml and subsequently used this for the inoculation of amphibian individuals.

### In vitro experiment

We tested the Bd growth inhibition capability of both *Xenorhabdus* CFCMs using optical density measurement, which is a semiquantitative test that provides more reliable results than agar diffusion tests (Bell et al. 2013). We prepared a 10-step twofold serial dilution starting with CFCM solution diluted with mTGhLY to 50% (v/v) on two flat bottom 96 well microplates (Orange Scientific, Braine-l’Alleud, Belgium) in a final volume of 50 µl. Then we added 50 µl zoospore suspension in 1% tryptone at a concentration of 10^7^ zsp/ml to the CFCM-containing wells (resulting in a final concentration of 5 × 10^6^ zsp/ml). Each plate also included three positive and three negative control wells containing 50 µl sterile mTGhLY and 50 µl intact or heat-killed (80 °C for 30 min) zoospore suspension in 1% tryptone. We incubated plates at 20 °C for 7 days in closed plastic boxes (30 × 15 × 10 cm). To prevent desiccation, we lined boxes with wet paper towels. After 7 days of incubation, we measured the optical density at 492 nm (OD_492_) using a Multiskan MS 352 microplate reader (Thermo Fisher Scientific, Waltham, USA).

### In vivo experiment

In March 2021 we set up 48 mesocosms by filling plastic boxes (85 × 57 × 51 cm) placed outdoors with 130 l of aged tap water and supplementing them with 50 g beech leaves for spatial complexity, and one litre of pond water containing bacterio-, phyto- and zooplankton. Four weeks later we added another 6 dl pond water to each mesocosm to boost zooplankton density and thereby reduce algal bloom. In April, we collected 200 eggs from each of four freshly laid egg strings of *B. bufo* from three localities near Budapest (Békás-tó: 47.5763° N, 18.869° E; Ilona-tó: 47.7135° N, 19.0402° E; Jávor-tó: 47.7138° N, 19.0196° E). We transported eggs to the Experimental Station Juliannamajor of the Plant Protection Institute, Centre for Agricultural Research located on the outskirts of Budapest (47.5479° N, 18.9349° E). We placed eggs of each clutch separately (families hereafter) into plastic boxes (32 × 22 × 16 cm) holding 0.7 l of reconstituted soft water (RSW; USEPA 2002) at a constant temperature of 16 °C and a light : dark cycle adjusted weekly to the conditions outside. Nine days after hatching, when all larvae reached development stage 25 (Gosner 1960), we released 50 individuals into each outdoor mesocosm (four mesocosms per family). The self-sustaining environments provided food and other nutrients for tadpoles without the need for any intervention until metamorphosis. For a schematic representation of the course of the experiment please see Fig S1.

Upon metamorphosis, when tadpoles reached development stage 42 (emergence of forelimbs), we monitored mesocosms daily and placed metamorphosing individuals into transparent plastic boxes (52 × 35 × 25 cm) containing 1.5 l of mesocosm water and covered with perforated lids. We tilted these boxes to provide a dry surface as well. When metamorphosis was complete (development stage 46; complete tail resorption), we haphazardly chose 25 toadlets from each family that metamorphosed on the same day and moved them into one rearing container per family (60 × 40 × 30 cm). These plastic containers were lined with 6 l of wet wooden soil, covered with a mixture of wet moss and leaf litter at a height of 6–8 cm. We covered containers with a perforated lid and placed them outdoors in an area shaded by trees. We watered containers weekly using RSW and fed juvenile toads *ad libitum* with springtails (*Folsomia* sp.) and small (2–3 mm) crickets (*Acheta* sp.) sprinkled with a 3:1 mixture of Reptiland 76,280 (Trixie Heimtierbedarf GmbH & Co. KG, Tarp, Germany) and Promotor 43 (Laboratorios Calier S.A., Barcelona, Spain) containing vitamins, minerals, and amino acids.

Fifty days after metamorphosis, we weighed the animals to the nearest mg (Ohaus Pioneer PA-213; Ohaus Europe Gmb, Nanikon, Switzerland), chose 8 medium-sized individuals from each family (N_total_ = 12 families × 8 replicate specimens = 96 individuals), and experimentally infected half of them. We performed experimental infections by placing the juvenile toads for five hours individually into sterile Petri dishes (diameter: 90 mm) containing 19 ml RSW and 1 ml liquid Bd culture in mTGhLY resulting in a final concentration of 50,000 zsp/ml (containing both zoospores and zoosporangia). The other half of the animals were sham-treated with the same amount of sterile mTGhLY. Individuals that were not selected for the experiment were released at their site of origin. Subsequently, we placed juvenile toads individually in covered opaque 2-L plastic boxes lined with wet paper towels as a substrate and a piece of egg carton as a shelter and reared them in the laboratory at 20.3 ± 0.3 °C (mean ± SD) and a light : dark cycle adjusted weekly to outdoor conditions. We arranged boxes on a shelf system in randomized spatial blocks, each containing all treatments from each infection status of a same family. We fed the juvenile amphibians with small crickets as described above.

One week after infection, we assessed the status of the skin microbiota of 12 experimentally infected individuals (‘positive control’ group) by swabbing the belly and hind legs (ten times each) using dry rayon-tipped swabs (MW100, Medical Wire & Equipment, UK). Swabs were stored at -20 °C until DNA isolation. Thereafter, we weighed these individuals (± 1 mg; Ohaus Pioneer PA-213), euthanized them using the “cooling then freezing” method (Shine et al. 2015) and finally preserved them in 96% ethanol at -20 °C until further processing. Subsequently, we exposed seven groups of 12 individuals of the remaining juvenile toads to seven treatments as described in Table 1. We decided to use *X. szentirmaii* CFCM diluted to 2 and 10% (v/v) based on a pilot experiment, aiming to apply treatments that are not yet harmful to amphibians but are still likely to be effective against Bd (for details see Supplementary text 1; Table S1). We performed these treatments by replacing juvenile toads from rearing boxes to sterile Petri dishes (55 mm diameter, Fig S2) and exposing them for three hours to either RSW, mTGhLY or CFCM solutions (for details see Table 1). We treated animals on four consecutive days. During the first treatment, we changed paper lining and shelter in the rearing boxes. On the last day of treatments, we swabbed, weighed, and preserved individuals as described above (Fig S1). Contaminated water and equipment were disinfected overnight with VirkonS (Johnson et al. 2003) and was disposed of following institutional guidelines on the treatment of dangerous waste.

**Table 1:** Details of the seven treatments applied on juvenile common toads

Bd-infection	Treatment	Exposure design	Treatment name	N
no	only RSW	7 ml RSW	RSW control	12
no	only mTGhLY	6.3 ml RSW + 0.7 ml mTGhLY	broth control	12
no	CFCM diluted to 2% (v/v)	6.3 ml RSW + 0.7 ml CFCM diluted to 20% (v/v) ^a^	low CFCM	12
no	CFCM diluted to 10% (v/v)	6.3 ml RSW + 0.7 ml pure CFCM	high CFCM	12
yes	only mTGhLY	6.3 ml RSW + 0.7 ml mTGhLY	Bd + broth control	12
yes	CFCM diluted to 2% (v/v)	6.3 ml RSW + 0.7 ml CFCM diluted to20% (v/v) ^a^	Bd + low CFCM	12
yes	CFCM diluted to 10% (v/v)	6.3 ml RSW + 0.7 ml pure CFCM	Bd + high CFCM	12

^a^CFCM was diluted with mTGhLY

### Assessment of infection intensity

We homogenized toe clips from the hind limbs of the preserved individuals, extracted DNA from samples using PrepMan Ultra Sample Preparation Reagent (Thermo Fisher Scientific, Waltham, Massachusetts, USA) according to previous recommendations (Boyle et al. 2004), and stored extracted DNA at -20°C until further analyses. We assessed infection intensity using real-time quantitative polymerase chain reaction (qPCR) following a standard amplification methodology targeting the ITS-1/5.8S rDNA region (ITS1-3 primer: 5’- CCTTGATATAATACAGTGTGCCATATGTC − 3’; 5.8 S primer: 5’- AGCCAAGAGATCCGTTGTCAAA − 3’; Boyle et al. 2004) on a BioRad CFX96 Touch Real-Time PCR System (BioRad Laboratories, Hercules, USA). To avoid PCR inhibition by ingredients of PrepMan, we diluted samples ten-fold with double-distilled water. We ran samples in duplicate, and in case of equivocal results, we repeated reactions in duplicate. If this again returned an equivocal result, we considered the sample to be Bd positive (Kriger et al. 2006). Genomic equivalent (GE) values were estimated from standard curves based on five dilutions of a standard (1000, 100, 10, 1, and 0.1 zoospore GE; provided by J. Bosch; Museo Nacional de Ciencias Naturales, Madrid, Spain). To assess whether cross-contamination occurred, we investigated one-third of individuals in each one of the uninfected treatment groups.

### Analysis of the skin microbiome

We randomly chose three samples from each treatment group, except for the ‘RSW control’ and the ‘low CFCM’ treatment groups, from which we chose five samples for the skin microbiome analyses. We analysed the bacterial community applying Illumina sequencing. DNA was isolated from the swabs using DNeasy® PowerSoil® Pro Kit (Qiagen) according to the manufacturer’s protocol and isolates were stored at -20°C until further use. For PCR amplification we used 16S rDNA specific primers: 341F forward primer with CS1 Illumina adapter sequence and 805R reverse primer with CS2 Illumina adapter sequence, and Phusion® DNA polymerase enzyme. We performed the PCR amplification three times on each sample. The final reaction volume was set to 20 µl. For a 1 µl template we measured 19 µl PCR mix, which contained 4 µl dNTP mix, 4 µl Phusion® HF Buffer, 0.2 µl CS1 341F forward primer (5’- CCTACGGGAGGCAGCAG − 3’), 0.2 µl CS2 805R reverse primer (5’- GACTACHVGGGTATCTAATCC − 3’), 0.4 µl bovine serum albumin (BSA), 0.2 µl Phusion® DNA polymerase and 10 µl PCR-grade water. In each PCR run, we included a positive (random sample that previously worked with the same primers) and a negative control. We applied the following heat profile: initial denaturation at 98 °C for 5 min followed by 25 cycles of denaturation at 95 °C for 30 s, annealing at 55 °C for 30 s and extension at 72 °C for 2 min. After the 25th cycle samples were kept at 72 °C for 10 min and then cooled to 4 °C. After each amplification, we checked the amplification success by visualizing PCR products on a 1% (w/v) agarose gel dissolved in TBE buffer (Fig S3). We ran samples in triplicates and pooled them after each amplification to reduce the influence of random bias that may occur in single reactions. We measured the DNA content of the pooled samples using a Qubit 2.0 Fluorometer (Invitrogen, Life Technologies, CA, USA). Finally, samples were sequenced on an Illumina MiSeq platform at the Research Technology Supply Facility (RTSF), Michigan State University (East Lansing, Michigan, USA).

### Statistical analyses

We ran all statistical analyses in R (version 4.0.5.). In case of the in vivo experiment, ‘RSW control’ and ‘positive control’ groups were only included in the analyses on the skin microbiota. We analysed the data of the in vitro Bd growth inhibitory potential by the two bacterial species separately using general linear models (LM) allowing the variances to differ among CFCM dilutions with the ‘varIdent’ function of the ‘nlme’ package. Models included OD_492_ as the dependent variable, and CFCM dilution and plate ID as categorical fixed factors. We compared each CFCM dilution step to the negative control by calculating pre-planned linear contrasts (Ruxton and Beauchamp 2008), while correcting the significance threshold for multiple testing using the false discovery rate (FDR) method (Pike 2011). We considered the means of two groups to differ when the relevant 84% confidence intervals (CIs) did not overlap (Payton et al. 2003; Julious 2004). We also identified the minimal inhibitory dilution (MID), which is the smallest dilution which still completely inhibits the growth of the test organism, i.e. the lowest dilution that’s 84% CIs still overlapped with the negative control.

Survival of juvenile toads was not analysed statistically, since only one death occurred (in the ‘Bd + broth control’ treatment). We averaged GE values obtained from qPCR runs for each sample and analysed resulting estimates of infection intensity in treatment groups that were experimentally exposed to Bd using generalized linear mixed models (GLMM) with negative binomial distribution and a log link function using the ‘glmmTMB’ package (Brooks et al. 2017). The model included CFCM dilution (0, 2, or 10%) as a categorical fixed factor and family nested in population as random factors.

We analysed body mass data using linear mixed models (LMM) with the ‘lme’ function of the ‘nlme’ package (Pinheiro et al. 2020). Since graphical model diagnostics indicated heterogeneous variances, we allowed the variances to differ among treatment groups with the ‘varIdent’ function of the ‘nlme’ package. We ran the initial model with infection status at the end of the experiment (yes or no) and CFCM dilution as categorical fixed factors and their interaction, the body mass of individuals measured at the start of the experiment as a numeric covariate, and family nested in population as random factors. We tested the effect of infection within each treatment group by calculating pre-planned linear contrasts (Ruxton and Beauchamp 2008), and corrected the significance threshold for multiple testing using the FDR method (Pike 2011).

During the experiment, we documented intensive skin shedding in infected individuals. To analyse the occurrence of shedding, we applied a generalized linear modelling procedure (GLM) with binomial distribution and logit link function containing infection status and CFCM dilution as categorical fixed factors. All tests were two-tailed, and we checked model fits in the case of all dependent variables by visual inspection of diagnostic plots. In all models containing interactions, we applied a backward stepwise removal procedure removing terms when P > 0.05 (Grafen and Hails 2002) to avoid problems potentially arising from the inclusion of non-significant terms (Engqvist 2005). We obtained statistics for removed variables by re-entering them one by one into the final model.

We processed raw sequencing data of the skin microbiome using the software Seed (version 2.1.1.; Větrovský et al. 2018). First, we joined forward and reverse sequences, then excluded poor quality sequences (mean quality value lower than 30) and sequences that contained ambiguous bases. We removed forward and reverse primers from the sequences. Singleton and short sequences (less than 360 nucleotides) were also excluded. We grouped sequences with at least 97% similarity into operational taxonomic units (OTUs) and removed chimeric sequences using Vsearch. We identified the representative sequences of the OTUs using the Basic Local Alignment Search Tool (BLAST) based on the ARB-SILVA database version 138 (Quast et al. 2013). We searched for archaeal, eukaryotic, and chloroplast sequences in our data and excluded them from the analysis. We set the number of sequences per sample to be the same as the number of sequences in the sample which contained the lowest number of sequences by random sampling. Then we assembled the OTU table which contains the number of OTUs per sample and calculated Chao1 diversity indices since this index is particularly useful for data sets skewed toward the low-abundance classes, as is likely to be the case with microbes (Hughes et al. 2001). To test whether Bd-infection or CFCM treatment and their interaction had a significant effect on the bacterial community living on the skin of the individuals we performed two-way permutational multivariate analysis of variance (PERMANOVA) based on Bray-Curtis similarity indices and non-metric multidimensional scaling (NMDS) analysis implemented in the software Past (version 4.05.). In the analyses on microbial species richness, we included the ‘positive control’ but excluded it from the PERMANOVA and NMDS analyses. To visualise the most abundant genera in treatment groups we used the R package ‘ampvis2’ (Andersen et al. 2018).

## Results

### In vitro experiment

**Fig. 1:**
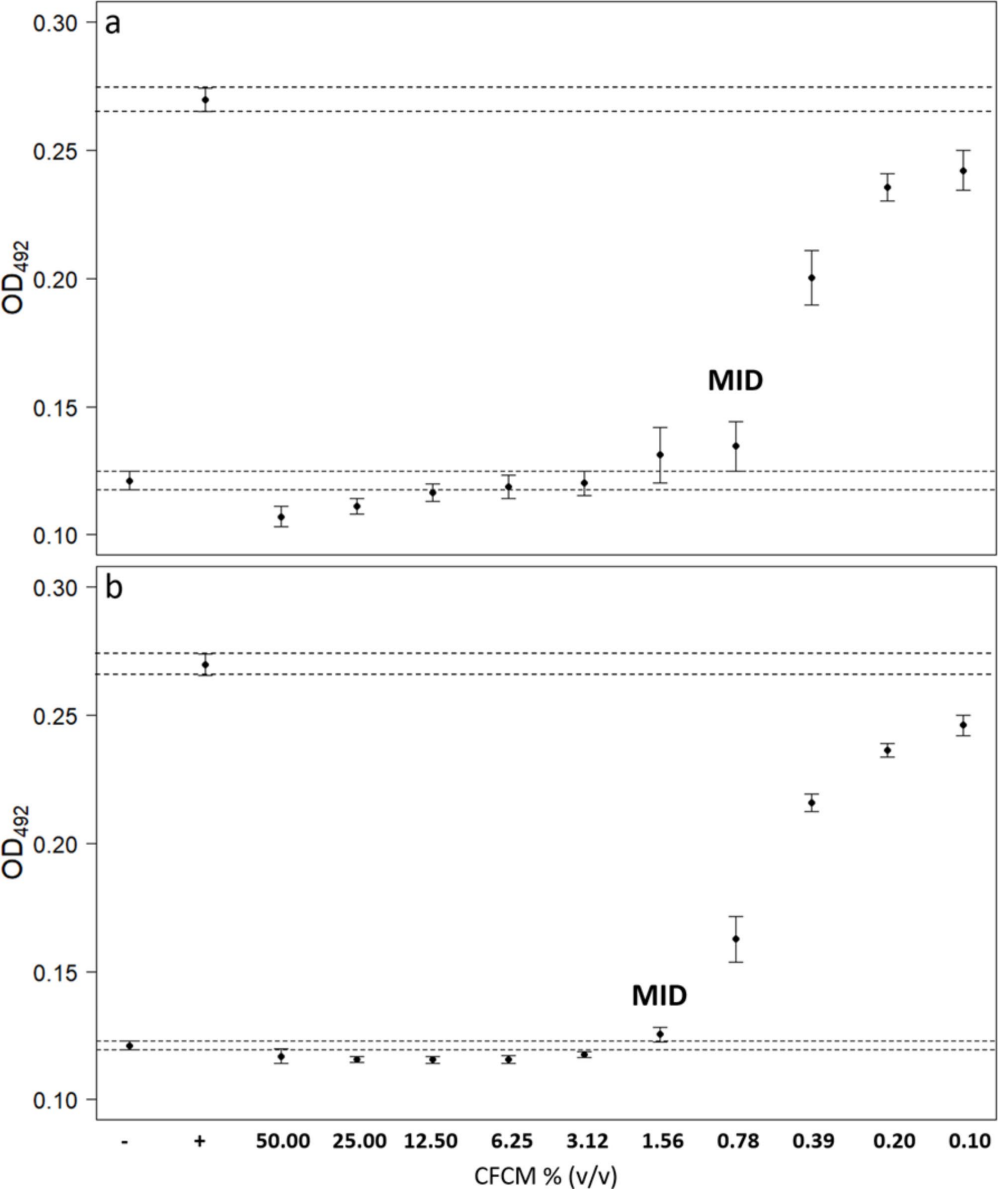
Bd-growth inhibition by serial dilutions of *X. budapestensis*** (a)** and *X. szentirmaii*** (b)** CFCM determined by measuring optical density at 492 nm (OD_492_; mean ± 84% CI). The negative control is represented by ‘-‘, the positive control by ‘+’. Dashed lines depict the 84% CI of the controls. Minimal inhibitory dilution (MID) indicates the lowest dilution with complete growth inhibition (84% CI still overlapping with that of the negative control)

The CFCM of both *Xenorhabdus* species significantly inhibited Bd growth (*X. szentirmaii*, LM: *F*_11, 78_= 386.74, *P* < 0.001; *X. budapestensis*, *F*_11,79_ = 751.36, *P* < 0.001) at all dilutions as neither of the respective 84% CIs of their mean OD_492_ values overlapped with the 84% CI of the positive control’s mean OD_492_ (Fig. 1). The MID of *X. szentirmaii* CFCM was 1.56% and that of *X. budapestensis* CFCM was 0.78% (Fig. 1; for results of pairwise comparisons see Table S2 and S3).

### In vivo experiment

**Fig. 2:**
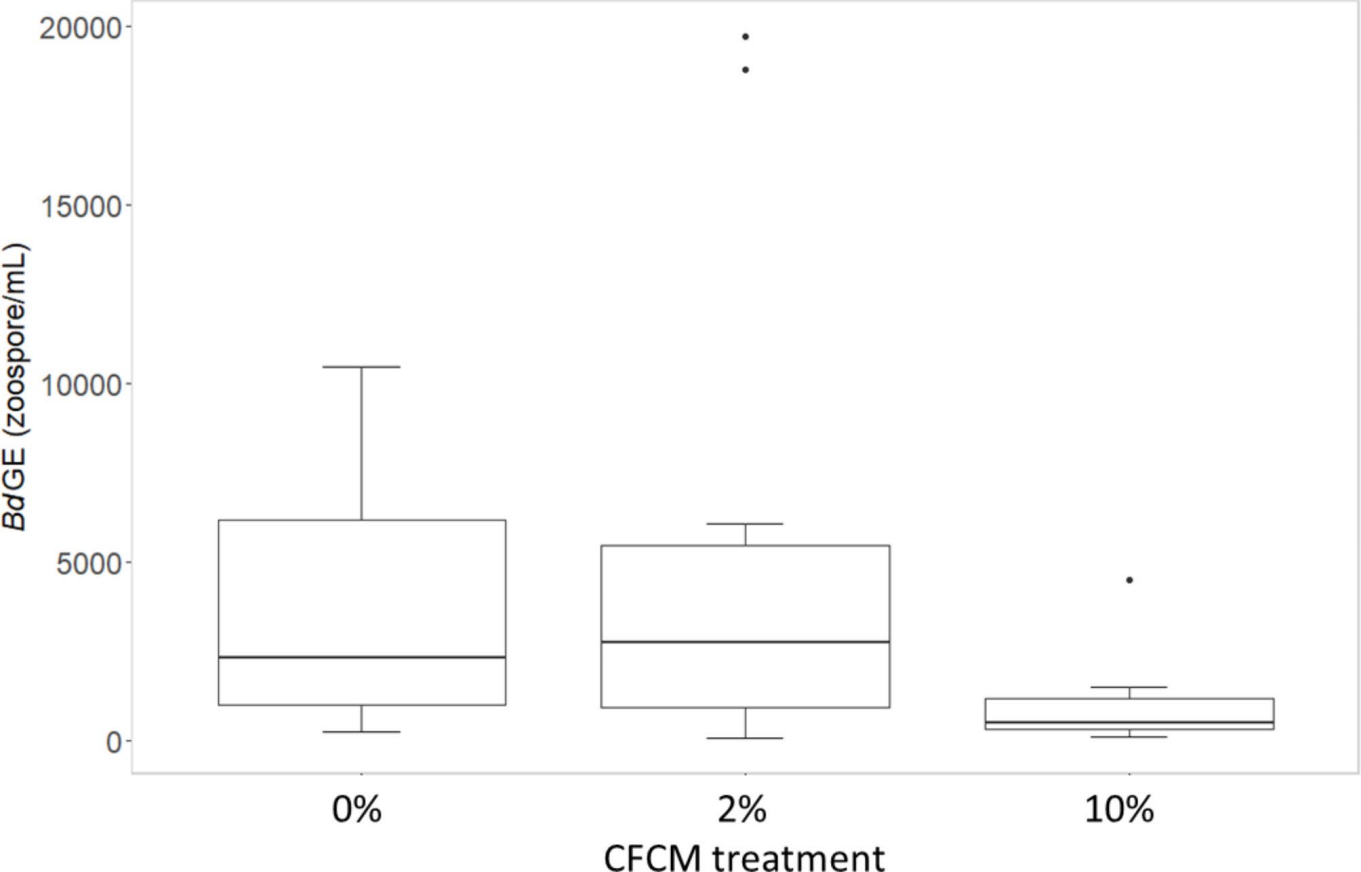
Infection load in Bd-exposed juvenile toads after treatment with sterile mTGhLY (0%), 2% or 10% CFCM of *X. szentirmaii*. Bold lines show medians, boxes show the interquartile range (IQR), bars represent ranges, dots indicate outliers (deviating from the boundary of IQR by more than 1.5 × IQR)

Survival was not affected over the course of the experiment by either treatment since all but one individual survived until the end of the study. We did not observe cross-contamination since all tested individuals from uninfected treatment groups remained Bd negative. Bd prevalence was 100% in all experimentally infected treatment groups. Infection intensity was 2417 ± 1539 GE (mean ± SD) per individual in the ‘positive control’ (treatment names refer for Table 1) right before the start of the CFCM treatments. Among CFCM treated infected groups, the ‘high CFCM’ (10% v/v) treatment resulted in significantly lower infection intensity compared to the ‘Bd + broth control’ treatment (Bd + broth control: 3666 ± 3468 GE [mean ± SD]; high CFCM: 984 ± 1201 GE [mean ± SD]; GLMM: *z* = -2.98, *P* = 0.003), however the ‘low CFCM’ (2% v/v) treatment did not affect infection intensity (*z* = 0.53, *P* = 0.59; Fig. 2). More concentrated *X. szentirmaii* CFCM (above 25% v/v) can be lethal for toads (Table S1).

**Fig. 3:**
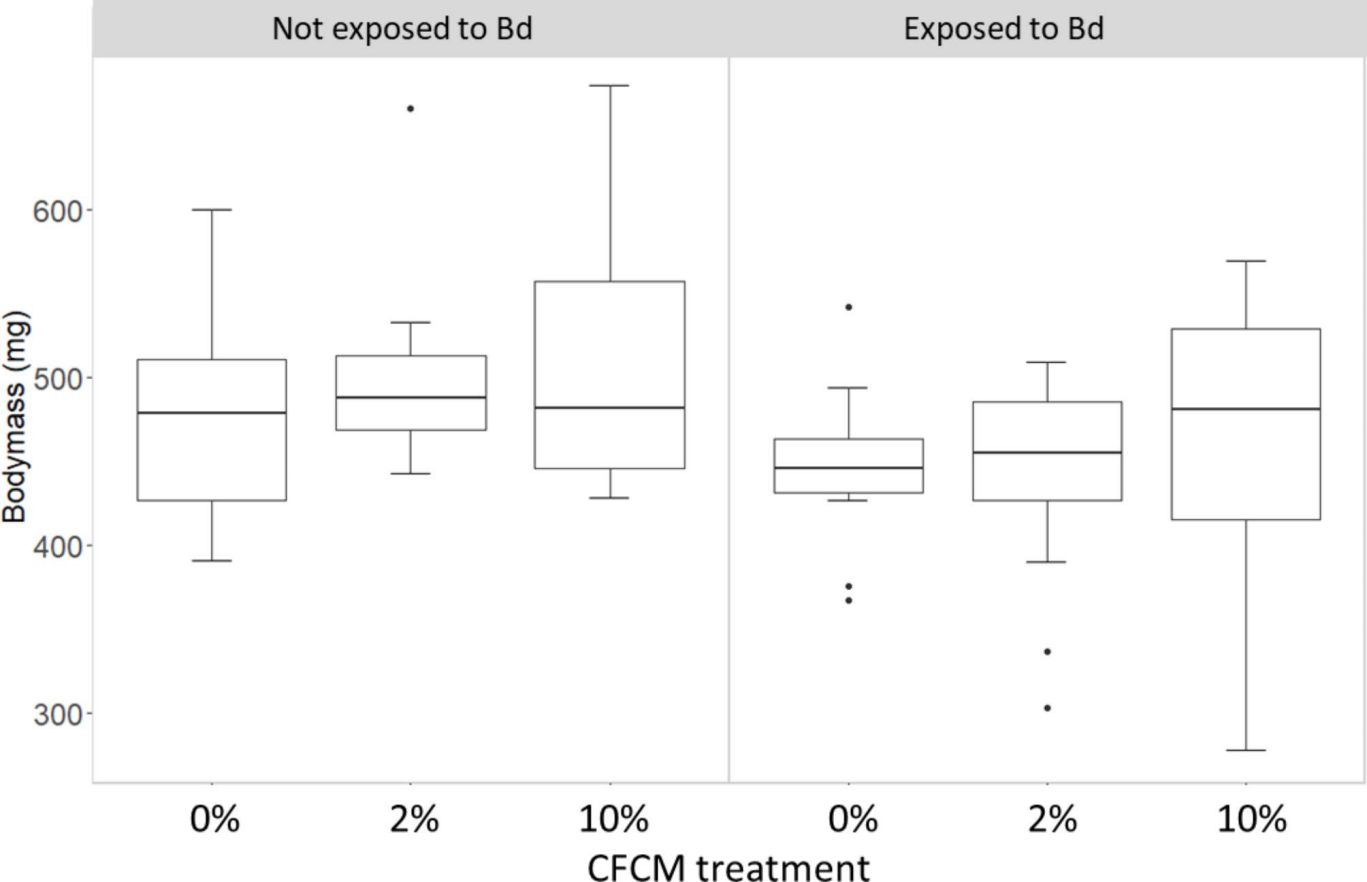
Body mass of juvenile toads at the end of the experiment in various treatment combinations. Bold lines show medians, boxes show the interquartile range (IQR), bars represent ranges, dots indicate outliers (deviating from the boundary of the IQR by more than 1.5 × IQR)

Body mass of the juvenile toads measured after treatments was negatively affected by Bd exposure (LMM: *F*_1,57_ = 12.32, *P* < 0.001), but treatment with CFCM had no significant effect on it, either alone (*F*_2,55_ = 0.92, *P* = 0.40) or in interaction with exposure to Bd (*F*_2,53_ = 0.17, *P* = 0.85; Fig. 3). Body mass measured at the termination of the experiment positively correlated with initial body mass measured at the start of the experiment (*B* = 0.8, SE = 0.13, *F*_1,57_ = 39.52, *P* < 0.001). Pairwise comparisons revealed that body mass of Bd-exposed individuals in the ‘broth control’ and ‘low CFCM’ was significantly lower compared to their non-infected counterparts (‘Bd + broth control’: t ratio = 2.25, df = 53, P = 0.028; ‘low CFCM’: t ratio = 2.82, df = 53, P = 0.007). However, body mass of individuals in the ‘high CFCM’ group did not differ between infected and non-infected individuals (t ratio = 1.34, df = 53, P = 0.188).

**Fig. 4:**
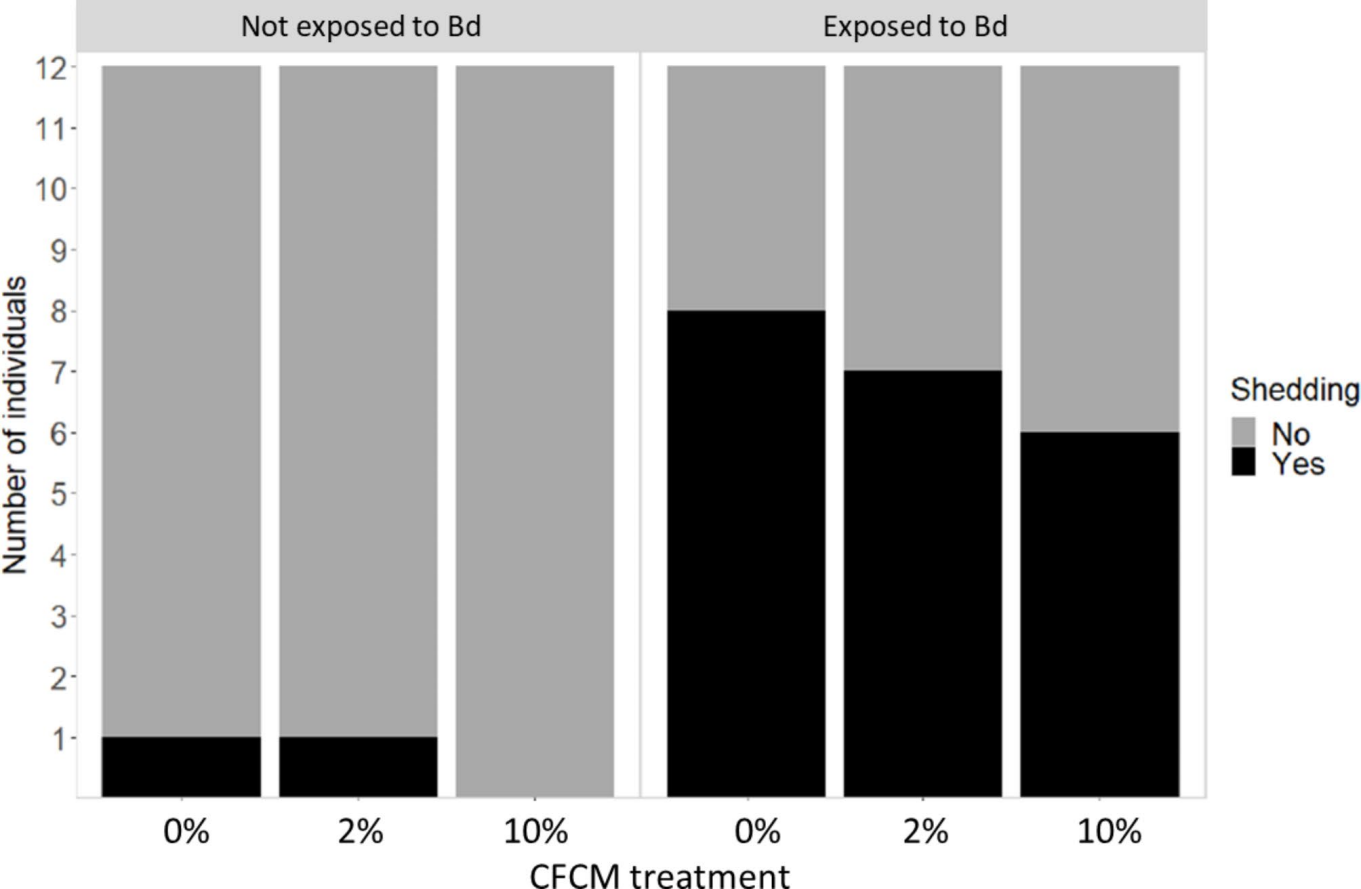
Frequency of skin shedding in various treatment combinations

We documented a significantly higher frequency of skin shedding among Bd-exposed individuals compared to controls (GLM: χ^2^ = 26.3, df = 1, *P* < 0.001), while exposure to CFCM did not significantly influence the propensity of animals to shed their skin (χ^2^ = 1.3, df = 2, *P* = 0.51; Fig. 4).

**Fig. 5:**
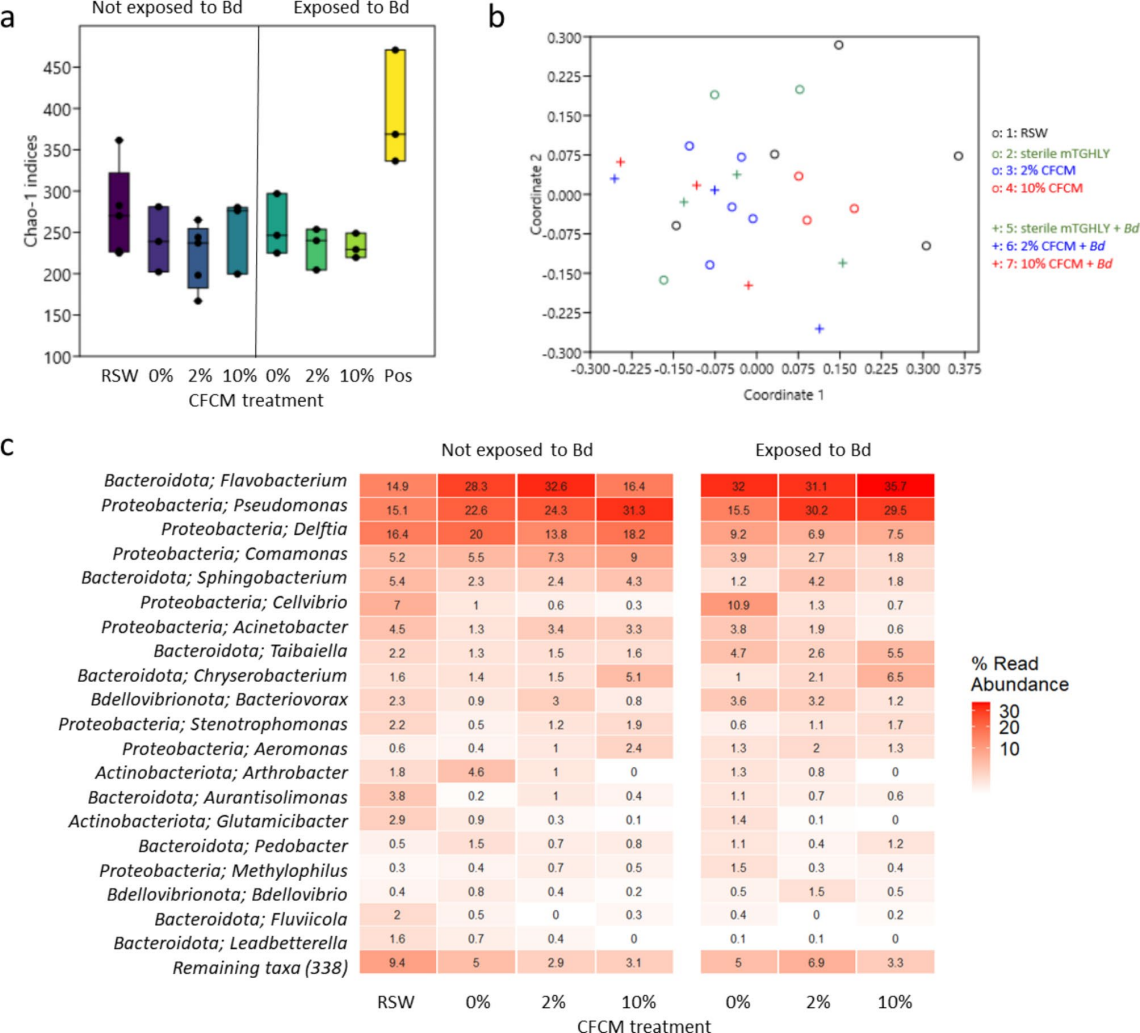
Result on skin microbiota analyses. **a:** Chao1 bacterial diversity indices of the samples of different treatment groups based on 16 S rDNA amplicon sequencing. Bold lines show medians, boxes show the interquartile range (IQR), bars represent ranges, dots are the original data points. RSW = ‘RSW control’, Pos = ‘positive control’. **b:** Similarity of bacterial community composition based on 16 S rDNA amplicon sequencing using NMDS ordination with Bray-Curtis distances. **c:** Most abundant bacterial genera in the skin microbiota of the juvenile toads based on 16 S rDNA amplicon sequencing listed by treatment groups. Shading indicates read abundance

Chao1 diversity indices reflecting species richness varied significantly among treatment groups (2-way ANOVA: *F*_4,27_ = 9.08, *P* < 0.001), where samples from the ‘positive control’ exhibited higher diversity of bacterial taxa than samples from any other treatment group, while the other treatment groups did not differ among each other (Table S4, Fig. 5a). Bd-infection had a marginally significant effect on the community structure of the skin microbiota (PERMANOVA: *F*_1,23_ = 1.73, *P* = 0.07), while the effect of CFCM treatment was non-significant both alone (*F*_6,17_ = 0.15, *P* = 0.57) and in interaction with Bd-infection (*F*_3,17_ = 0.35, *P* = 0.94). NMDS analysis (stress value: 0.27) did not reveal any systematic pattern, ordination appeared to be arbitrary (Fig. 5b). Visual inspection of the relative abundance of the most abundant bacterial genera in the skin microbiota of the juvenile toads based on 16 S rDNA amplicon sequencing or focussing only on the three most abundant bacterial genera (*Flavobacterium*, *Pseudomonas*, and *Delftia*) also did not reveal striking differences among treatments (Fig. 5c).

## Discussion

The present study is the first to demonstrate Bd growth inhibition by EPB metabolites. In the in vitro experiment both the *X. szentirmaii* and the *X. budapestensis* CFCM were highly effective in inhibiting Bd growth, and this effectiveness was confirmed using the *X. szentirmaii* CFCM in the in vivo experiment. At the same time, measurable adverse effects of the treatment on juvenile common toads did not surface either in terms of lowered survival, lowered body mass or perturbed skin microbiome.

We found that dilutions of both *Xenorhabdus* CFCMs (as low as 1.56 and 0.78%) fully halted Bd growth in vitro, while CFCM diluted to 0.1% still showed some level of inhibition. This antimicrobial activity of *X. szentirmaii* CFCM against Bd is much higher compared to former studies that described MID values of 40 and 10% against bacterial pathogens of humans (Ozkan et al. 2019) and plants (Vozik et al. 2015). Consequently, and because EPB CFCMs are already effectively used against several bacterial and fungal pathogens, the application of *X. szentirmaii* CFCM has great potential in the fight against chytridiomycosis.

The most important finding of this study is that the ‘high CFCM’ treatment (10% dilution) had a striking effect on Bd infection load in experimentally infected juvenile toads. Although the ‘low CFCM’ treatment (2% dilution) did not lower infection intensity in the in vivo experiment, treatment with the 10% dilution of *X. szentirmaii* CFCM reduced infection load by 73%. Thus, a complete clearance of the infection was not reached, which would prevent the re-growth of the pathogen within individuals and its further spread to other hosts. Nonetheless, the observed high level of infection intensity suppression is also very promising because a complete clearance of infection is not essential for the prevention of mortalities: former studies reported no clinical signs of infection and no mortalities at low levels of infection (Vredenburg et al. 2010; Cheng et al. 2011). Furthermore, amphibian populations can coexist with Bd and maintain large population sizes if infection intensities remain low (Rowley and Alford 2013). Finally, co-existence with enzootic Bd may allow for adaptation to the disease via the spread of alleles providing increased resistance or tolerance on the level of populations (Bataille et al. 2015; Savage and Zamudio 2016; Voyles et al. 2018) and via immunization on the level of individuals (Ramsey et al. 2010; McMahon et al. 2014). To enhance efficiency, CFCM may be applied for extended times or on a larger number of consecutive days, but this would also increase the chances or severity of negative side-effects (see below).

The in vitro MID value of *X. szentirmaii* CFCM was 1.56% but the ‘low CFCM’ treatment (2%) proved to be ineffective against Bd in the in vivo experiment. These seemingly contradictive results may be partly explained by differences in the duration of exposure, which was continuous for one week in the in vitro experiment versus only three hours on four consecutive days in the in vivo experiment. The experimental microenvironment differed a lot as well: Bd cells were directly exposed to CFCM in the homogeneous mTGhLY in the in vitro experiment, while in the in vivo experiment Bd thalli were to some extent protected inside the keratinized epithelial cells of the amphibian skin. Hence, our study stresses that although in vitro experiments can provide valuable baseline data, in vivo experiments are indispensable when testing the effectiveness of new methods of disease mitigation.

The CFCMs we used likely contain more than one antifungal agent, but it was not our aim to determine their chemical identity and concentration. In this study only the supernatant of the bacterial cultures was used, in which exoenzymes such as chitinases can be also present. Since autoclaved *Xenorhabdus* CFCMs exerted similar antimicrobial and antifungal effects as the native ones in other experiments involving various pathogens (but not Bd; Fodor et al. 2022, 2023), the activity of the CFCM is most likely effected by thermostable secondary metabolites and not by exoenzymes. Fabclavines are highly effective and thermostable antifungal compounds which are known to be present in *Xenorhabdus* CFCM (Cimen et al. 2021), but whether these were the compounds which were active against Bd remains to be verified. How the antifungal activity of the EPB CFCMs and their constituents relates to that of metabolites produced by bacteria associated with amphibian skin (Brucker et al. 2008a, b; Myers et al. 2012; Woodhams et al. 2018) remains unknown because we investigated the activity of all metabolites produced by EPBs in their entirety, while the studies using skin bacteria focussed on individual antimicrobial metabolites. Future studies may verify the anti-Bd activity of fabclavines by exposing Bd or Bd-infected animals to CFCM, to fabclavine only, or to CFCM from a fabclavine production deficient *Xenorhabdus* mutant.

Although the survival of toads can sharply decrease upon infection with Bd (Garner et al. 2009; Bielby et al. 2015; but also see Ujszegi et al. 2021), we found that survival of juvenile toads was not affected by infection with Bd, perhaps because the Bd isolate we used co-occurs with the sampled toad population (also see Kásler et al. 2022). We documented a significant reduction in body mass after eleven days of Bd exposure. Other studies have also shown that exposure to Bd can lead to a decrease in body mass (Parris and Cornelius 2004; Blaustein et al. 2005; Hanlon et al. 2015; Kásler et al. 2022), one of the best predictors of fitness in juvenile amphibians (Semlitsch et al. 1988; Altwegg and Reyer 2003). In the present study, we also documented that shedding was more frequent in the Bd-infected treatment groups than in the non-infected groups. This is in accordance with earlier observations from Berger et al. (2005), Voyles et al. (2009) and Martel et al. (2011). Excessive skin shedding most likely serves as a defence mechanism aiming to remove the infected outer epithelial cell layers (Becker and Harris 2010; Meyer et al. 2012). Finally, PERMANOVA detected a marginally significant effect of Bd-infection (assuming a p-value threshold of 0.05) on the skin bacterial community due to Bd-infection itself, while Chao1 indices did not differ between infected and uninfected juvenile toads, which is in accordance with results of an earlier study (Jani and Briggs 2014).

We expected to detect some harmful effects of CFCM treatment, because EPBs produce toxins that harm the insect that is colonised by the host EPNs and contribute to its death (Forst et al. 1997). However, we did not find this. Treatment with 10% *X. szentirmaii* CFCM did not cause any surplus mortality nor did it affect body mass adversely. Moreover, the negative effect of Bd exposure on body mass was abolished by the ‘high CFCM’ treatment. CFCM treatment did not affect the frequency of skin shedding in non-infected groups, but it also did not prevent infection-induced skin shedding in the infected groups. Finally, the lack of an effect of CFCM treatment on the skin microbiota we found on the juvenile toads was surprising because of its wide-spectrum antimicrobial activity, including several bacterial taxa. Possibly, the dilutions we applied were too high to affect microbial growth under in vivo conditions in a complex microbial community. Alternatively, changes may not have surfaced right after the treatment and may have appeared later. However, decrease of microbial diversity in all groups compared to the ‘positive control’ group suggests that detectable differences in microbial communities manifest very rapidly (Fig. 5a). Alternatively, the limited sample sizes may have prevented the detection of subtle differences. However, striking immediate effects of CFCM treatment on skin microbiota were clearly absent, and several microbial taxa with anti-Bd properties remained on the juvenile toads’ skin after the CFCM treatments (Supplementary text 2). In sum, *X. szentirmaii* CFCM diluted to 10% does not appear to harm juvenile common toads. Nonetheless, it remains to be determined whether more concentrated dilutions of EPB CFCM cause harmful effects and to what extent amphibian species and life-stages differ in their susceptibility to CFCM treatment.

We propose that the application of antifungal metabolites without the need for the establishment of probiotic bacteria on the skin of amphibians may be a good solution that would bridge the problems arising from bioaugmentation or the application of conventional antifungals such as itraconazole or amphotericin B (Garner et al., 2009; Holden et al. 2014). Antifungal metabolites of *Xenorhabdus* bacteria are highly potent candidates because they are easy to cultivate, cheap to produce, highly effective and exceptionally stable even at high temperatures (Fodor et al. 2022). These results of our pioneering study are the first to suggest that EPB CFCMs can be applied effectively and safely to treat Bd-infected amphibians at least under laboratory conditions, where re-application can be performed easily. Currently, 180 endangered amphibian species subsist only thanks to captive breeding programs (Kueneman et al. 2022), and amphibians are increasingly maintained in captivity for research, hobby, public display, and food production (Hadfield and Whitaker 2005; Densmore and Green 2007). These activities require an easily applicable, non-invasive, chemical-free, and relatively cheap mitigation method to halt chytridiomycosis outbreaks and prevent the reintroduction of infected individuals into natural habitats. Our results suggest that the antifungal metabolites of *Xenorhabdus* bacteria may prove suitable for this task. At the same time, however, this approach also has some limitations, which have to be thoroughly tested and considered before application. Metabolites must be re-applied either until complete clearance or until an effective immune response is mounted by the host. Furthermore, as CFCM contains a culture medium, it can promote the growth of other bacteria (especially in case of prolonged CFCM exposure), which are not sensitive to the contained metabolites and some of these may be facultative or obligate pathogens. Finally, even subtle changes in the skin microbiome may influence the fitness of amphibian hosts. Consequently, to provide the necessary knowledge base, studies will need to identify the antifungal components present in the EPB CFCM and assess their concentration, as well as optimize the method of application to maximize effectiveness. Furthermore, testing for potential malign long-term effects of treatment with CFCM, and investigating its applicability to other amphibian species and pathogens would also be important. Ultimately, CFCM treatment may prove to be an effective and safe approach to the mitigation of chytridiomycosis and perhaps other diseases of amphibians and other vertebrates as well.

In summary, we demonstrated for the first time that metabolites produced by entomopathogenic bacteria of the genus *Xenorhabdus* can halt Bd growth in vitro already at very low concentrations. We also documented that these metabolites can be safely applied to amphibian juveniles and that the treatment resulted in drastically lowered infection intensities. Hence, our results support the idea that *Xenorhabdus* metabolites may very well be used in the mitigation of chytridiomycosis in live amphibians. Future studies on this system will likely prove fruitful and will contribute to exploiting the surprising effectiveness of EPB metabolites against this devastating amphibian disease.

### Electronic supplementary material

Below is the link to the electronic supplementary material.

Supplementary Material 1

## Data Availability

All data used in the analyses will be available from Figshare Repository upon publication. 10.6084/m9.figshare.21229439.
