# Supplementary material for: Metabolites of Xenorhabdus bacteria are potent candidates for mitigating amphibian chytridiomycosis
Source: AMB Express. 2023 Aug 24;13:88. doi: 10.1186/s13568-023-01585-0 (PMC10449739; doi:10.1186/s13568-023-01585-0)
Supplement: Supplementary file 1 — Supplementary Material 1 [file 13568_2023_1585_MOESM1_ESM.docx]

AMB Express

Supplementary materials to:

Metabolites of *Xenorhabdus* bacteria are potent candidates for mitigating amphibian chytridiomycosis

János Ujszegi^1,2*^, Zsófia Boros^1,3^, András Fodor^3^, Balázs Vajna^4^, Attila Hettyey^1,2^

^1^Department of Evolutionary Ecology, Plant Protection Institute, Centre for Agricultural Research, Eötvös Loránd Research Network, Budapest, Hungary

^2^Department of Systematic Zoology and Ecology, Eötvös Loránd University, Budapest, Hungary

^3^Department of Genetics, Eötvös Loránd University, Budapest, Hungary

^4^Department of Microbiology, Eötvös Loránd University, Budapest, Hungary

*Corresponding author

e-mail: ujszegi.janos@gmail.com

tel.: +36-1-3918607

fax: +36-1-3918653

ORCID ID: 0000-0002-6030-0772

**
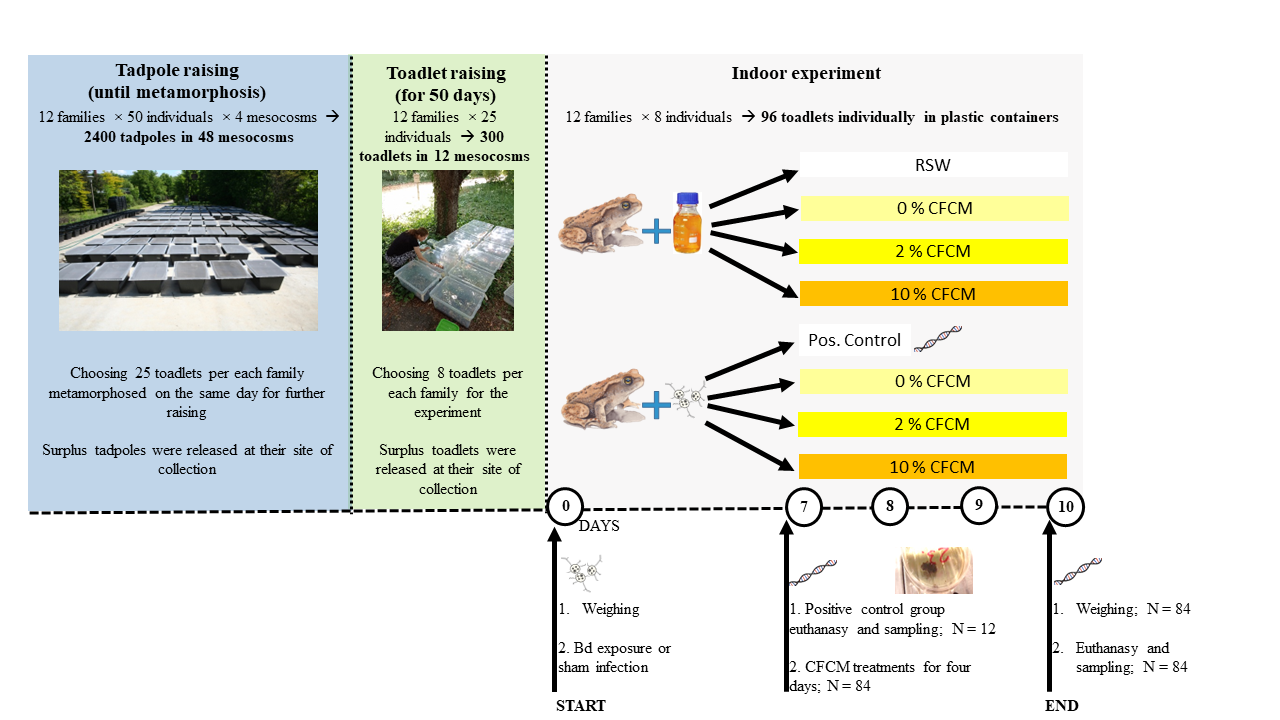
**

**Figure S1** A schematic illustration of the course of the experiment. Toad paintings were made by Bálint Bombay.

**Supplementary text 1: Finding the optimal CFCM dilution to be used on live animals**

We performed pilot tests before the main experiment to assess the toxicity of CFCMs of both *Xenorhabdus* species and to find CFCM dilutions appropriate for *in vivo* disinfection. We randomly assigned 6 specimens to each of the seven treatment groups: treatment 1: only mTGhLY, treatment 2: pure *X. budapestensis* CFCM, treatment 3: *X. budapestensis* CFCM diluted to 50% (v/v), treatment 4: *X. budapestensis* CFCM diluted to 25%, treatment 5: pure *X. szentirmaii* CFCM, treatment 6: *X. szentirmaii* CFCM diluted to 50%, treatment 7: *X. szentirmaii* CFCM diluted to 25%. CFCM was diluted in sterile mTGhLY. We pipetted 7 ml CFCM solution or sterile medium into plastic Petri dishes (55 mm diameter) and kept individuals inside for five hours (Fig S2). We inspected for the signs of toxicity (abnormal body posture and vomiting) and registered deaths.

While mTGhLY itself had no effect on the juvenile toads, treatments 2-5 were lethal for all individuals, and two out of the six animals died in treatment 6. We repeated treatment 1, 6 and 7 in the next two days with the rest of the individuals, but reduced exposure time for three hours per day. By the end of the third day, only one individual survived treatment 6, and we found vomited gastric content (Fig S2) in the Petri dish at 66% of them overall. In contrast, all individuals survived treatment 7 (25% *X. szentirmaii* CFCM) without any sign of toxicity. Results are summarized in Table A1. Hereupon we decided to use *X. szentirmaii* CFCM only with less than 25% CFCM content.


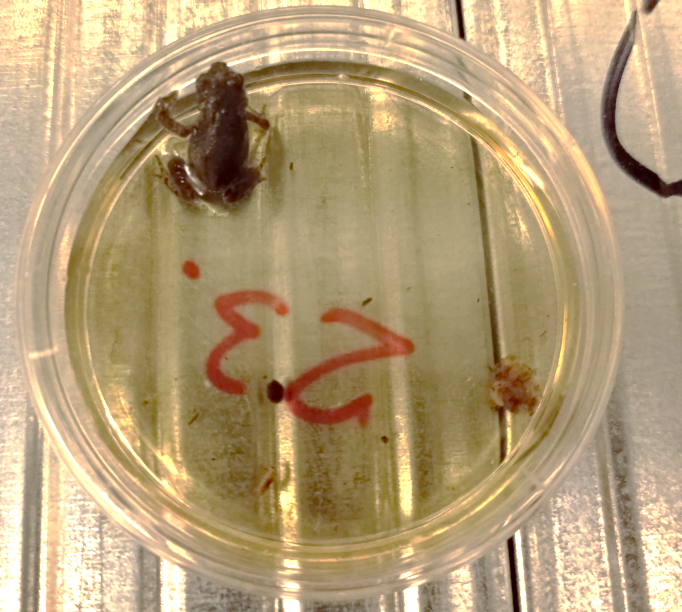


**Figure S2** Rearing conditions during CFCM treatment. Vomited gastric content consist of dead larval crickets can be seen on the right side (pilot treatment 6: 50% *X. szentirmaii* CFCM).

**Table S1** Survival of juvenile toads during the three days of the preliminary experiment

| Treatments | | Died within… | | | Overall survival (%) |
| --- | --- | --- | --- | --- | --- |
|  |  | First day | Second day | Third day |  |
| 1 | mTGhLY | 0 | 0 | 0 | 100 |
| 2 | 100% *X. budapestensis* CFCM | 6 | - | - | 0 |
| 3 | 50% *X. budapestensis* CFCM | 6 | - | - | 0 |
| 4 | 25% *X. budapestensis* CFCM | 0 | 6* | - | 0 |
| 5 | 100% *X. szentirmaii* CFCM | 6 | - | - | 0 |
| 6 | 50% *X. szentirmaii* CFCM | 2 | 1 | 2 | 17 |
| 7 | 25% *X. szentirmaii* CFCM | 0 | 0 | 0 | 100 |
| *Survived the first day, but died next day before repeating treatments | | | | | |

**
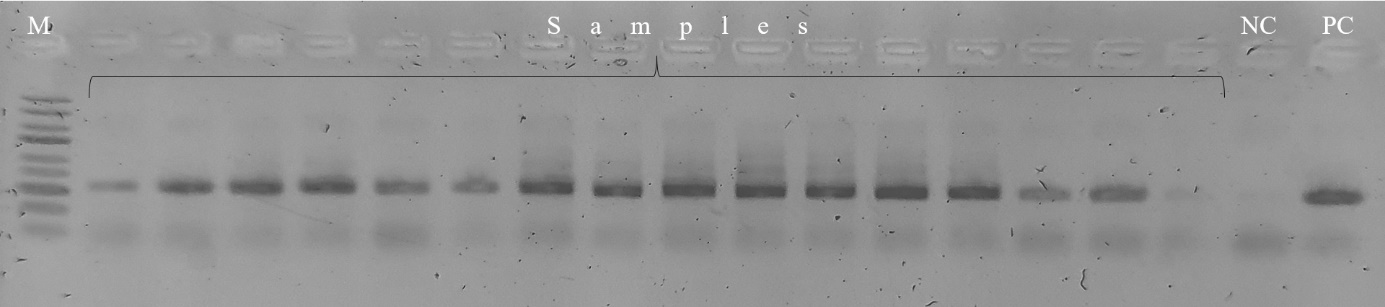
**

**Figure S3** A gel image of the PCR products amplified for the microbiota analyses with a set of samples. M: marker, NC: negative control, PC: positive control

**Table S2** Results of pre-planned comparisons comparing mean OD_492_ value of Bd growth in case of each dilution of *X. szentirmaii* CFCM to the mean OD_492_ values of the negative control. Linear contrasts (*c*), associated standard errors (SE), *t*-values and *P*-values adjusted using the FDR method are reported. Significant differences (P < 0.05) from mean OD_492_ of the negative control are highlighted in bold.

| CFCM Dilution (%) | c | SE | *t* | *P* |
| --- | --- | --- | --- | --- |
| **0.10** | **0.12583** | **0.00257** | **48.90** | **< 0.001** |
| **0.20** | **0.11746** | **0.00299** | **39.32** | **< 0.001** |
| **0.39** | **0.10033** | **0.00613** | **16.37** | **< 0.001** |
| **0.78** | **0.04746** | **0.00784** | **6.05** | **0.001** |
| 1.56 | 0.00808 | 0.00424 | 1.91 | 0.102 |
| 3.12 | -0.00217 | 0.00191 | -1.13 | 0.281 |
| **6.25** | **-0.00467** | **0.00165** | **-2.82** | **0.023*** |
| **12.5** | **-0.00467** | **0.00159** | **-2.94** | **0.023*** |
| **25.0** | **-0.00579** | **0.00145** | **-3.99** | **0.017*** |
| 50.0 | -0.00417 | 0.00204 | -2.05 | 0.076 |
| *Significantly lower OD_492_ than that of the negative control | | | | |
|  |  |  |  |  |

**Table S3** Results of pre-planned comparisons comparing mean OD_492_ value of Bd growth in case of each dilution of *X. budapestensis* CFCM to the mean OD_492_ values of the negative control. Linear contrasts (*c*), associated standard errors (SE), *t*-values and *P*-values adjusted using the FDR method are reported. Significant differences (P < 0.05) from mean OD_492_ of the negative control are highlighted in bold.

| CFCM Dilution (%) | c | SE | *t* | *P* |  |
| --- | --- | --- | --- | --- | --- |
| **0.10** | **0.12098** | **0.00512** | **23.64** | **< 0.001** |  |
| **0.20** | **0.11433** | **0.00385** | **29.72** | **< 0.001** |  |
| **0.39** | **0.07908** | **0.00692** | **11.43** | **< 0.001** |  |
| 0.78 | 0.01333 | 0.00635 | 2.10 | 0.061 |  |
| 1.56 | 0.00996 | 0.00705 | 1.41 | 0.198 |  |
| 3.12 | -0.00104 | 0.00354 | -0.29 | 0.769 |  |
| 6.25 | -0.00242 | 0.00338 | -0.72 | 0.525 |  |
| 12.5 | -0.00479 | 0.00281 | -1.71 | 0.127 |  |
| **25.0** | **-0.01004** | **0.00262** | **-3.83** | **0.001*** |  |
| **50.0** | **-0.01404** | **0.00310** | **-4.53** | **< 0.001*** |  |
| *Significantly lower OD_492_ than that of the negative control | | | | | |
|  |  |  |  |  |  |

**Table S4** Results of 2-way ANOVA Tukey post-hoc tests on Chao1 diversity indices.

| Tukey post hoc P values | |  |  |  |  |
| --- | --- | --- | --- | --- | --- |
|  | RSW control | broth control | low CFCM | high CFCM | positive control |
| RSW control |  | 0.86 | 0.32 | 0.75 | < 0.001 |
| broth control |  |  | 0.86 | 0.99 | < 0.001 |
| low CFCM |  |  |  | 0.95 | < 0.001 |
| high CFCM |  |  |  |  | < 0.001 |
| positive control |  |  |  |  |  |

**Supplementary text 2: Skin microbial composition on juvenile toads**

We found a diverse microbial community on juvenile common toads, with the most abundant OTUs belonging to the genera *Flavobacterium*, *Pseudomonas*, and *Delftia*. Flavobacteria are practically omnipresent in aquatic habitats and moist soil (Bernardet & Bowman 2006) and are recognized to be opportunistic pathogens of lower vertebrates, causing flavobacteriosis in amphibians (Densmore & Green 2007). Pseudomonads can be the etiological agents of bacterial dermatosepticaemia (Densmore & Green 2007) while they are known to be inhibitory to Bd and other fungal pathogens(Harris et al. 2006; Flechas et al. 2012; Myers et al. 2012), just as members of the genus *Delftia* (Park et al. 2014). In general, individuals in the ‘positive control’ group hosted more diverse microbial communities than those in other treatment groups, possibly because these animals were euthanized four days earlier and thereby may have lost fewer bacterial taxa under the artificial conditions of the laboratory (Kueneman et al. 2022). Note that the ‘positive control’ group is considered as a positive control for the Bd infection (thus not a positive control from the point of view of the microbiota).

Literature cited

Bernardet JF, Bowman JP. 2006. The Genus *Flavobacterium*. The Prokaryotes **7**:481–531.

Densmore CL, Green DE. 2007. Diseases of amphibians. ILAR journal / National Research Council, Institute of Laboratory Animal Resources **48**:235–254.

Flechas S V., Sarmiento C, Cárdenas ME, Medina EM, Restrepo S, Amézquita A. 2012. Surviving chytridiomycosis: Differential anti-*Batrachochytrium dendrobatidis* activity in bacterial isolates from three lowland species of Atelopus. PLoS ONE **7**.

Harris RN, James TY, Lauer A, Simon MA, Patel A. 2006. Amphibian pathogen *Batrachochytrium dendrobatidis* is inhibited by the cutaneous bacteria of amphibian species. EcoHealth **3**:53–56.

Kueneman J et al. 2022. Effects of captivity and rewilding on amphibian skin microbiomes. Biological Conservation **271**:109576.

Myers JM, Ramsey JP, Blackman AL, Nichols AE, Minbiole KPC, Harris RN. 2012. Synergistic inhibition of the lethal fungal pathogen *Batrachochytrium dendrobatidis*: The combined effect of symbiotic bacterial metabolites and antimicrobial peptides of the frog *Rana muscosa*. Journal of Chemical Ecology **38**:958–965.

Park ST, Collingwood AM, St-Hilaire S, Sheridan PP. 2014. Inhibition of *Batrachochytrium dendrobatidis* caused by bacteria isolated from the skin of Boreal Toads, *Anaxyrus* (*Bufo*) *boreas boreas*, from Grand Teton National Park, Wyoming, USA. Microbiology Insights **7**:1–8.
